# Pioneer of Cardiothoracic Surgery - Luiz Tavares da Silva

**DOI:** 10.21470/1678-9741-2023-0046

**Published:** 2023-11-08

**Authors:** Ricardo de Carvalho Lima, Leonardo Pontual Lima, Mozart Augusto Soares de Escobar, José Ricardo Lagreca de Sales Cabral, José Aécio Fernandes Vieira, Guilherme Tavares da Silva Maia

**Affiliations:** 1 Department of Surgery, Faculdade de Ciências Médicas, Universidade de Pernambuco (UPE), Recife, Pernambuco, Brazil; 2 Department of Surgery, Universidade Federal de Pernambuco (UFPE), Recife, Pernambuco, Brazil; 3 Department of Thoracic and Cardiovascular Surgery, Pronto-Socorro Cardiológico Universitário de Pernambuco Prof. Luiz Tavares (PROCAPE), Universidade de Pernambuco, Recife, Pernambuco, Brazil; 4 Department of Surgery, Universidade Federal do Rio Grande do Norte (UFRN), Natal, Rio Grande do Norte, Brazil; 5 Department of Surgery, Hospital Universitário Oswaldo Cruz, Recife, Pernambuco, Brazil; 6 Department of Surgery, Grupo Fernandes Vieira (GFV), Recife, Pernambuco, Brazil; 7 Department of Surgery, Instituto de Medicina Integral Professor Fernando Figueira (IMIP), Recife, Pernambuco, Brazil

**Keywords:** Pioneer, Cardiothoracic, Surgery, Luiz Tavares Da Silva, Cardiac Surgery

## Abstract

Luis Tavares revolutionized cardiac surgery, always bringing the most modern instruments and equipment from his travels to England - surgical forceps, scissors, scalpels, etc. He always insisted that he was not just a thoracic surgeon, for his work extended over a wide field and created three important cardiac surgery centers which promoted a great development of cardiology. He carried out the first open heart surgery (atrial septal defect) employing extracorporeal circulation and closure of a ventricular septal defect with deep surface hypothermia of north and northeast Brazil. He promoted an intense scientific exchange program between Recife and England, resulting in significant advances in medicine, and participated directly in the creation of HEMOPE), leading to radical changes and improvements in blood therapy in the whole country. The PROCAPE, inaugurated in 2006, was the result of the cardiac center created by him in early 1970 at Hospital Oswaldo Cruz and can be considered the second largest public-university cardiology center in Brazil. He is thus widely regarded as an outstanding name in medicine in the 20^th^ century and one of the fathers of modern cardiac surgery in Brazil.

## INTRODUCTION

**Table:** 

Abbreviations, Acronyms & Symbols	
FCM	= Faculdade de Ciências Médicas
HCR	= Hospital Centenário de Recife
HEMOPE	= Fundação de Hematologia e Hemoterapia de Pernambuco
HOC	= Hospital Oswaldo Cruz
ICR	= Instituto de Cardiologia do Recife
INAMPS	= Instituto Nacional de Assistência Médica da Previdência Social
PROCAPE	= Pronto-Socorro Cardiológico Universitário de Pernambuco Prof. Luiz Tavares
UFPE	= Universidade Federal de Pernambuco
UK	= United Kingdom
UPE	= Universidade de Pernambuco
USA	= United States of America
VSD	= Ventricular septal defect

### Biography

**Fig. 1:**
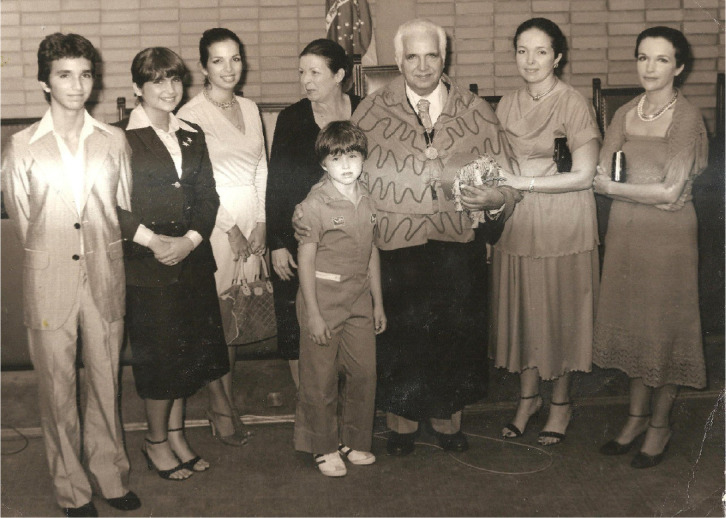
April 17^th^, 1979, Luiz Tavares and his family during the Professor Emeritus Ceremony at the Universidade Federal de Pernambuco when he deserved this title for having achieved the highest degree in the exercise of its academic activity.

Luiz Carvalho Tavares da Silva came from a traditional Brazilian family. He was born in the city of Recife (Pernambuco, Brazil) on April 16^th^, 1916, and died on June 27^th^, 1994. His father, Arsenio Luiz Tavares da Silva, was a professor of general surgery. His mother, Joana Miranda de Carvalho, was a housewife from the State of Bahia. He had three siblings: Manuel, Maria, and João. In 1948, he married Maria Dulce Coimbra de Almeida Brennand, and they had seven children: Dulce, Francisca, Joana, Izabel, Antonia, Luiz, and Manuel (Figure 1)^[1-12]^.

### High School, Medical School, and Postgraduate Studies

Luiz Tavares had a privileged education at Colégio São Bento in the city of São Paulo, having completed his medical studies at the Universidade de São Paulo. (After graduating in 1939, he returned to Recife to work with his father in the Hospital Centenário de Recife (HCR). His post-graduation in thoracic surgery was in England, specifically in Leeds, Oxford, and London. In England, he had the opportunity to work with famous thoracic and cardiovascular surgeons such as Sir Philip Allison (his personal friend), Sir Russel Brock, and Sir Holmes Saylors, among others.

### Medical Career

Luiz Tavares’s ambition from the beginning was to become a leader in surgery and he lost no time in pursuing his goal. After a series of junior appointments as a general surgeon, he took the decision to specialize in the chest area and become a thoracic surgeon. He went first to Leeds to train with Professor Philip Allison. After went back to Recife, he returned to work at HCR. His main surgical contribution there was to set a practical example. He always insisted that he was not just a thoracic surgeon, for his work extended over a wide field. His surgical technique was outstanding, and he was immediately recognized as a leader in his specialty. In the operating theatre, he combined boldness and originality in conception with meticulous care in execution. He spent much time instructing nurses so as to ensure an optimal teamwork. His unit resembled a small, closely-knit family, and his surgical team at HCR afforded a great impulse to cardiac surgery, performing surgical procedures such as closed mitral commissurotomy, ductus arteriosus ligation, Blalock-Taussig shunt, resection of coarctation of the aorta, and resection of aortic aneurysms. As regards aortic surgery, several techniques were performed at that time: aneurysmal sac wiring, wrapped aorta with cellophane paper tapes, aneurysm resection, and a nylon graft interposition using surface hypothermia at 28°C. Despite HCR being considered the leading heart surgery unit in Pernambuco state, he decided to leave that hospital, owing to its lack of investment in high-cost equipment, as he had learnt that it was not possible to maintain one’s leadership in thoracic and cardiac surgery without a high investment in equipment and human resources. The existing difficulties led him to move to a new heart center in Hospital Dom Pedro II, together with his surgical team comprising, Mauro Arruda, Milton Lins, and Eugênio Albuquerque.

### Cardiac Institute

**Fig. 2:**
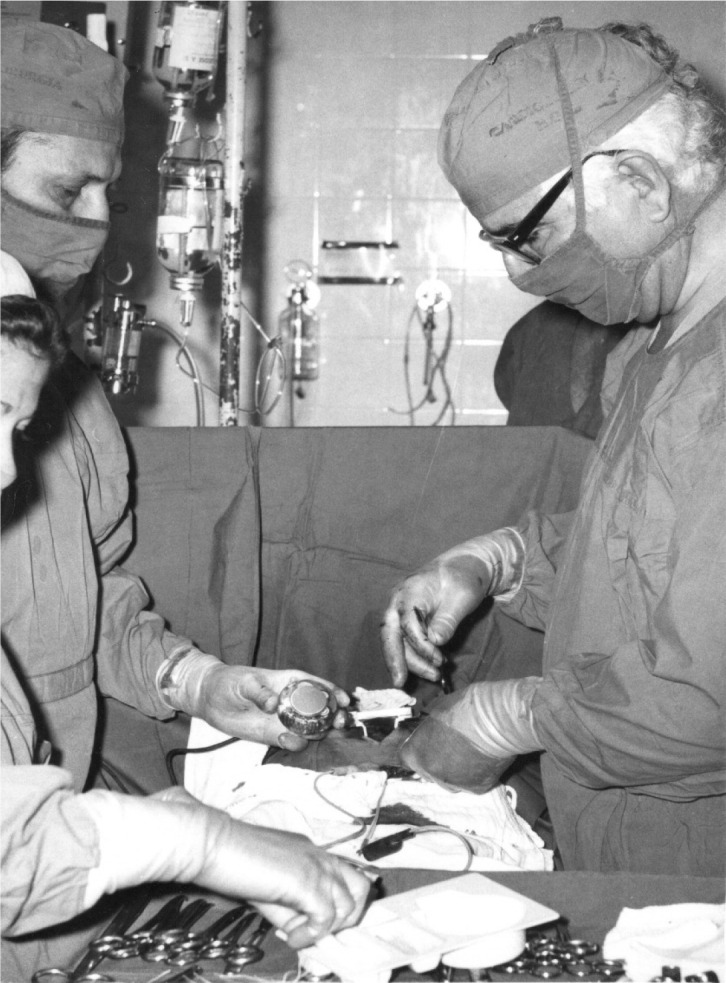
Luiz Tavares performing a pacemaker implant surgery at the Instituto de Cardiologia/Hospital Dom Pedro II, Recife, Brazil.

In 1956, Fernando Simões Barbosa, a physician and pioneering cardiologist in northeast Brazil, created a heart center named Instituto de Cardiologia do Recife (ICR), located in Hospital Dom Pedro II and affiliated to the Universidade Federal de Pernambuco (UFPE). The creation of this cardiac center was possible thanks to the financial support of the Rockefeller Foundation, the CAPES, and the CNPq. The ICR played a key role in the growth of cardiology in the northeast region of the country, which by that time had acquired a national reputation. The collaboration between physicians and surgeons working as partners in the same place resulted in major advances in local cardiology, with a constantly renewed and expanding team, whose scientific production was recognized throughout Brazil. At the ICR, health care and scientific activities were carried out with great intensity (Figure 2).

When Luiz Tavares moved to the ICR, despite considerable initial resistance to invest in expensive equipment, he succeeded in creating a first-class department. With direct financial support from the Dean of the UFPE and his own financial resources, he went to London and purchased a complete surgical cardiac unit from the Genyto-Urinary Company. The operating table was the type used at Brompton Hospital, London, and was very sophisticated at that time. The surgical drapes used during surgery were made from Irish linen in a green color. The quality of the human resources was also a matter of concern to him, especially nursing professionals, exemplified by his hiring of an outstanding French nurse, Eliane Leveque. He also sent countless young doctors to study abroad, thereby creating a particularly strong connection between England and Recife. The ICR was already highly developed at that time, with an outpatient clinic, a clinical pathology laboratory, a department of graphical methods, a hemodynamic laboratory, vectorcardiography, phonocardiography, an operating room, and a postoperative intensive care. Regular clinical meetings were held with all the staff to discuss the best way to treat a patient. The ICR was a cradle of scientific exchange, its facilities being visited by numerous distinguished individuals in the field of cardiology, such as, among many others, Prof. Hugo Fillipozzi (Brazil), Prof. Euryclides de Jesus Zerbini (Brazil), Sir Philip Allison (United Kingdom), Enrique Cabrera (Mexico), David Watson (United States of America), Peter Sleight (United Kingdom), the Queen’s doctor), Aulf Gunning (United Kingdom), Emmanuel Lee (United Kingdom), Marian Ionescu (United Kindgdom), and Christopher Lincoln (United Kingdom).

### Pioneer in Thoracic and Cardiovascular Surgery

**Fig. 3:**
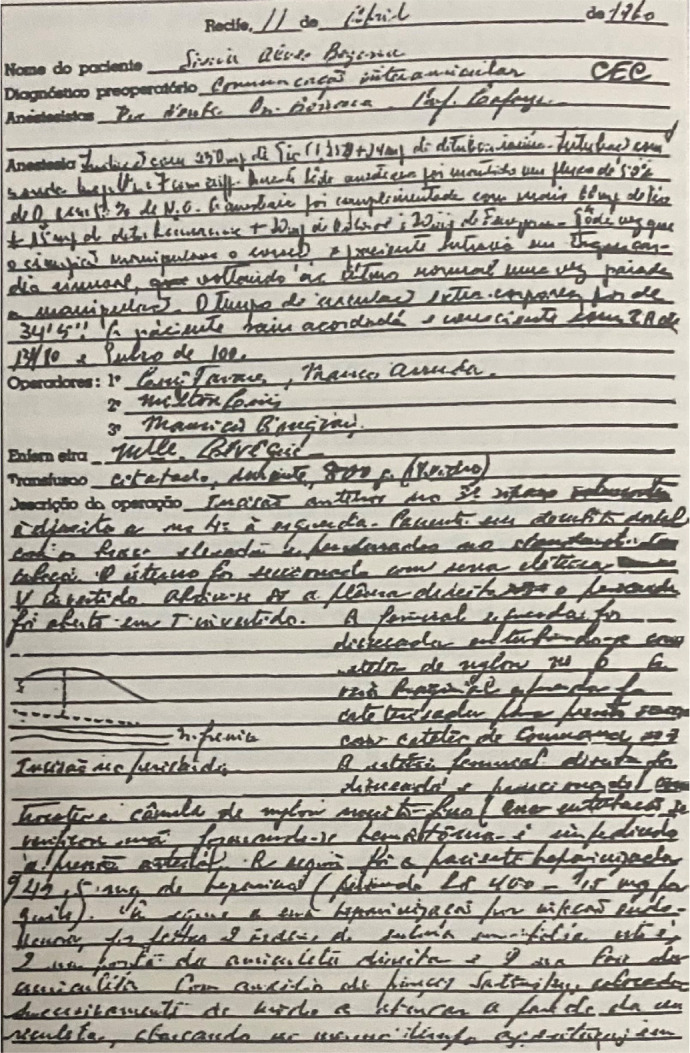
Operative description of the first surgery procedure for closure of the atrial septal defect with cardiopulmonary bypass in north-northeast Brazil.

**Fig. 4:**
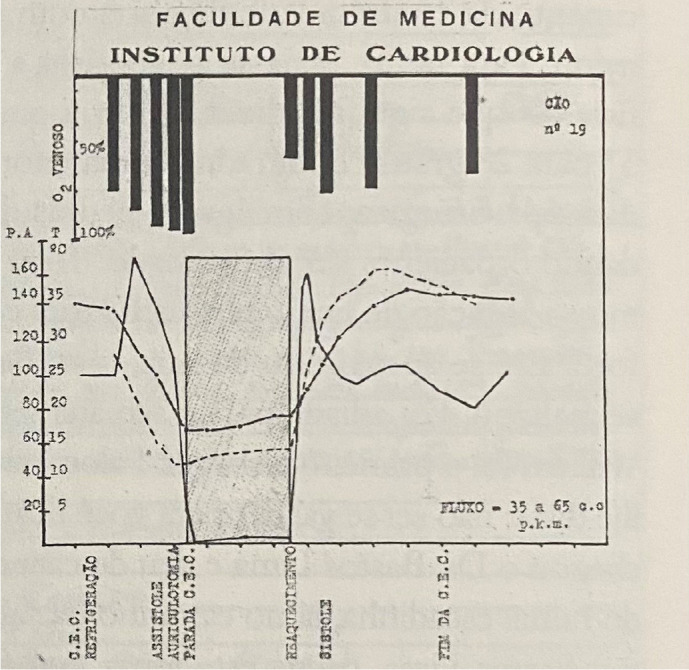
Results of the experimental study of deep hypothermia in 20 dogs before the first patient was operated on with a cardiopulmonary bypass.

In the north and northeast regions of Brazil, the first cardiac surgery under direct vision was performed in ICR by Luiz Tavares and his assistants Mauro Arruda, Milton Lins, and Mauricio Bouqvar. In January 1960, a patient with pulmonary stenosis was successfully operated on using surface hypothermia and total occlusion of the venae cavae. Three months later, the first patient was operated on for cardiopulmonary and cardiac arrest (Figure 3). The patient operated on bypass had a diagnosis of atrial septal defect, and the surgery was a success. Seven years after the first operation - performed by John Gibbon in the USA - in the world, Luiz Tavares and his surgical team achieved this feat in Recife. Recife was the third city in Brazil performing open heart surgery with extracorporeal circulation, following only São Paulo and Rio de Janeiro. Records show that at that time, even Italy, a European country, had not yet performed its first open heart surgery with the aid of a heart-lung machine. The pump machine used by Luiz Tavares was a Pemco, with rollers and a Kay-Cross disk oxygenator. One year later, on April 7^th^, 1961, Luiz Tavares performed the first correction of ventricular septal defect (VSD) with deep surface hypothermia and total circulatory arrest for 32 minutes. After this achievement, the ICR became an active, productive center, responsible for the training of countless clinical cardiologists and surgeons, publishing several papers in various scientific journals. Prior to the first open heart surgery in 1960, exhaustive experimental work on extracorporeal circulation and deep hypothermia in dogs had already been carried out (Figure 4).

### Academic Life

In 1956, Luiz Tavares replaced his father in the chair of the 2^nd^ Surgical Clinic of the Faculdade de Medicina do Recife through a public examination. He was considered an outstanding candidate and remained there until his retirement in 1978. During his academic life, he held two full professorships. He defended the thesis: “Surgical medical study of Manson’s schistosomiasis” in the competitive examination contest for the academic post of “Docente Livre” at the Surgical Clinic of the Faculdade de Medicina do Recife. This was the first publication on hepatosplenic schistosomiasis in Brazil. He also defended the thesis “Diagrammatic hernia, esophagitis and peptic ulcer of the esophagus”. During his professional life, he presented numerous scientific papers in congresses and produced a large number of scientific publications.

Since 1950, in each year there was a large number of students who passed the medical selection exam but were not admitted to the medical school due to a limited number of places for medicine. In response to the appeals of young people who were unable to enter higher medical education, Luiz Tavares joined a group of medical professors who decided to establish a new medical school named Faculdade de Ciências Médicas (FCM). He became a founder member, subsequently its chairman. At present, 72 years after the creation of FCM, more than 9,000 physicians have graduated. He became Full Professor of Thoracic Surgery at the two public universities existing in Recife at that time: the UFPE and the Universidade de Pernambuco. In the year 2000, Ricardo de Carvalho Lima replaced Luiz Tavares as Full Professor of Thoracic Surgery of FCM/UPE, as result of a public examination, and he occupies this post to the present. Continuing the work started by Luiz Tavares at FCM, Ricardo Lima created the first medical residency program in cardiothoracic surgery at UPE. This residency program came to fill an important gap in the training of cardiac surgeons, since the traditional residency program in cardiothoracic surgery at UFPE had been discontinued in the early 1990s. Also in 2019, a new residency program in pediatric cardiology was created by Ricardo Lima. From 1970 to 2023, hundreds of doctors have been trained.

### Hospital Oswaldo Cruz

The Hospital Oswaldo Cruz (HOC) has its origins in Hospital Santa Ageda, created to treat patients during a smallpox epidemic in 1884. Between 1951 and 1954, a thoracic surgeon, Joaquim Cavalcanti, made enormous contributions to Brazilian medicine, pioneering the first surgery to correct a congenital heart disease (systemic-pulmonary shunt - Blalock-Taussig surgery) and surgery for acquired heart disease (mitral valve repair) in the Brazilian north and northeast regions. He died prematurely but had already planted the seeds of heart surgery in the State of Pernambuco.

In the early 1970s, when the ICR ceased to exist due to reforms implemented by the Brazilian Federal Government, Luiz Tavares turned his attention to HOC and inaugurated a new heart center. In August 1972, an agreement for the purpose of establishing a new center of cardiology was signed between HOC and the Instituto Nacional de Assistência Médica da Previdência Social (INAMPS). This new cardiology unit was linked to the FCM and from then on, local cardiology achieved great progress. Luiz Tavares (FCM), Antonio Figueira (FCM), and Alcedo Gomes (INAMPS) were responsible for the abovementioned agreement. The new heart center continues existing to the present day, training countless clinicians and surgeons. In 1975, that agreement resulted in the creation of the region’s first coronary unit, its first public cardiology emergency hospital, and the first specialization course in cardiology.

Luiz Tavares once again used his personal prestige in obtaining resources to rebuild the surgical center and the intensive care unit for the exclusive use of cardiology patients, with wards for both adults and children. Two operating rooms were built with a high degree of sophistication, with electric tables, a gasometer, and invasive monitoring. The postoperative intensive care unit was directly connected to the operating room to facilitate patient transportation. In 1971, the first surgery was performed there by Milton Lins on a patient with rheumatic mitral stenosis, who underwent a digital mitral commissurotomy, and the surgical team acquired great experience of this technique in Brazil. Prestigious surgeons had the opportunity to operate at HOC, including Adib Jatene (1972) and Christopher Lincoln (1977). The HOC cardiac center operated uninterruptedly for 35 years (1971-2006) when in 2006 the unit moved to new hospital facilities at the Pronto-Socorro Cardiológico Universitário de Pernambuco Prof. Luiz Tavares (PROCAPE).

### Pronto-Socorro Cardiológico Universitário de Pernambuco Prof. Luiz Tavares

**Fig. 5:**
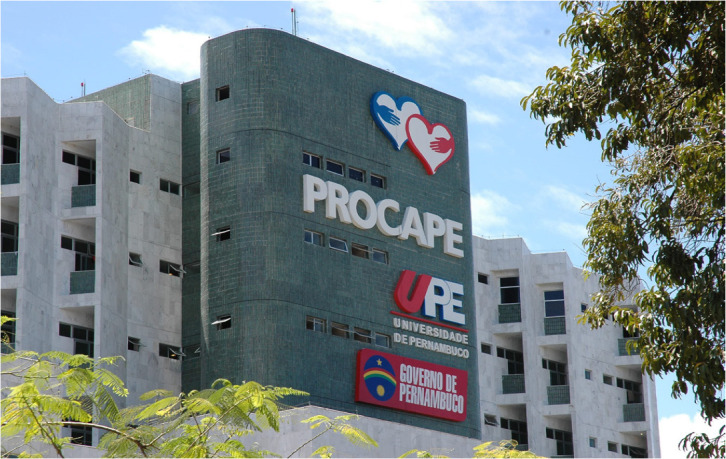
Pronto-Socorro Cardiológico Universitário de Pernambuco Prof. Luiz Tavares (PROCAPE), Recife, PE.

In 2006, after the Luiz Tavares’ great contribution to cardiology, another professor, Enio Lustosa Cantarelli, understanding the need to promote the expansion of cardiology, using public funds, conceived, built, and inaugurated a new cardiology center. In honor of Professor Tavares, the new school hospital was named Prof. Luiz Tavares (or PROCAPE). This new hospital is a public teaching hospital in cardiology and part of the health complex of UPE with 220 beds (Figure 5). From 1974 to 2022, 48,380 heart surgeries were performed (24,026 at HOC and 24,354 at PROCAPE). This teaching hospital offers 299 vacancies for regular curriculum health internships and 95 vacancies for medical and multidisciplinary residency training, in addition to being a major research center.

### Creation of Fundação de Hematologia e Hemoterapia de Pernambuco

In 1977, there was no national policy on hematology in Brazil. A political decision by the state government and the leadership of two doctors, Luiz Tavares and Antônio Figueira, led to the creation of the Fundação de Hematologia e Hemoterapia de Pernambuco (HEMOPE) and this became the first public blood center in Brazil. The aim was to improve the quality of hematology and hemotherapy in Brazil, and this quality improvement involved three goals: creating the discipline of hematology at FCM, developing scientific research, and producing blood products industrially. The project was completed in 2011, when the third objective was achieved with the inauguration of Hemobrás, with the aim of producing blood products on an industrial scale. HEMOPE was responsible for the radical change in national policy on hemotherapy under the direction of the Ministry of Health. Today the hematology and hemotherapy system in Brazil is a source of pride and one of the safest systems in the whole world, arising from a very well-structured project led by Luiz Tavares, and can be considered the embryo of modern hemotherapy in Brazil. The entire structure of HEMOPE was based on the French system, which is considered one of the best systems in the world.

### Medical Exchange Training with England

When Luiz Tavares completed his training in Leeds, he returned to Recife, having established during this time, a solid friendship with Professor Philip Allison that would last for the rest of his life. Allison had enormous prestige throughout the world and was responsible for the development of the heart-lung machine in England, having influenced the professional career of Luiz Tavares. It is fair to say that much of the innovative work performed during the Allison era can be credited to his first assistant, Alfred James Gunning, who moved with him from Leeds to Oxford. Gunning and Allison were pioneers in heart valve homografts and pig xenografts, techniques subsequently used in many centers around the world. Luiz Tavares’ friendship with those two brilliant English surgeons established a solid basis for the medical exchange program between Oxford and Recife. In 1970, Luiz Tavares consulted the British Council in Recife and hired David Randall as an English teacher for the FCM students, with the aim of improving their knowledge of the English language aiming at a future training of these doctors in England. This led to countless medical doctors from Recife going to England for training, contributing in an unusual way to Brazilian medicine. Among some of these doctors are: José Aécio Vieira, Antonio Figueira, Ney Cavalcanti, Caio Souza Leão, Sávio Barbosa, Edgar Victor, Luciano Raposo, Alcides Bezerra, Carlos Moraes, Hildo Azevedo, Marcelo Azevedo, Catarina Cavalcanti, Fernando Cavalcante, Fátima Militão, Paulo Almeida, Francisco Bandeira, Amaro Andrade, Cláudio Lacerda, Cícero Rodrigues, George Teles, Pedro Arruda, Ricardo de Carvalho Lima, Guido Corrêa de Araújo, Gustavo Gibson, Leila Beltrão, Leandro Araújo, Tércio Barcelar, Geraldo Furtado, Ricardo Pernambuco, Marcelo Maia, Renato Della Santa, Eugenia Cabral, Gustavo Caldas and others.

### Sports and hobbies

**Fig. 6:**
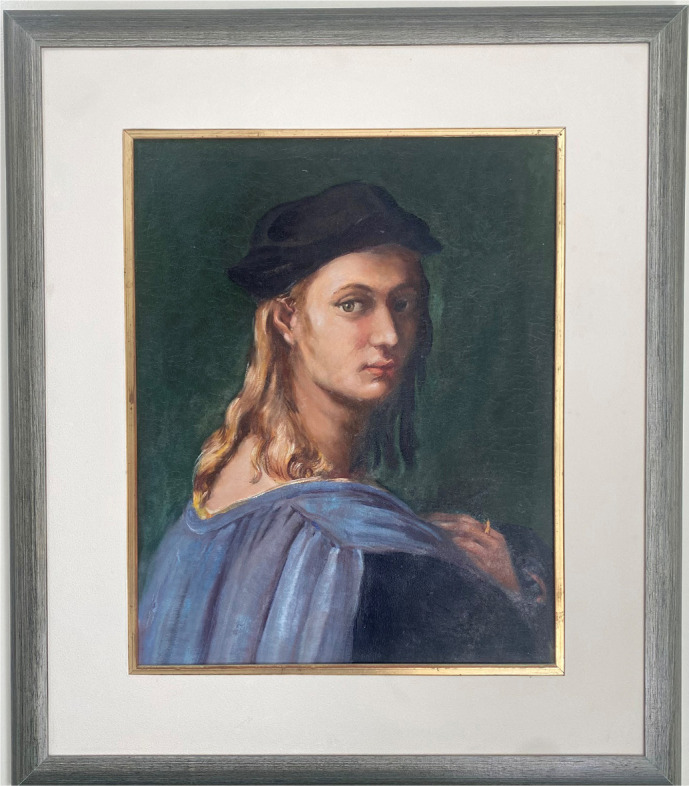
Painting by Luiz Tavares donated by family members to Pronto-Socorro Cardiológico Universitário de Pernambuco Prof. Luiz Tavares (PROCAPE), Recife, PE.

**Fig. 7:**
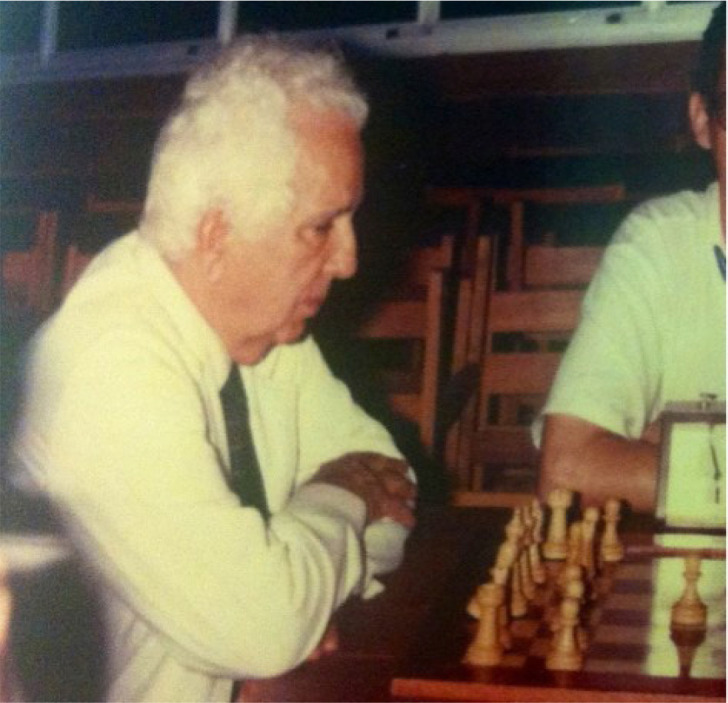
Luiz Tavares devoting himself to chess, his favorite hobby.

**Fig. 8:**
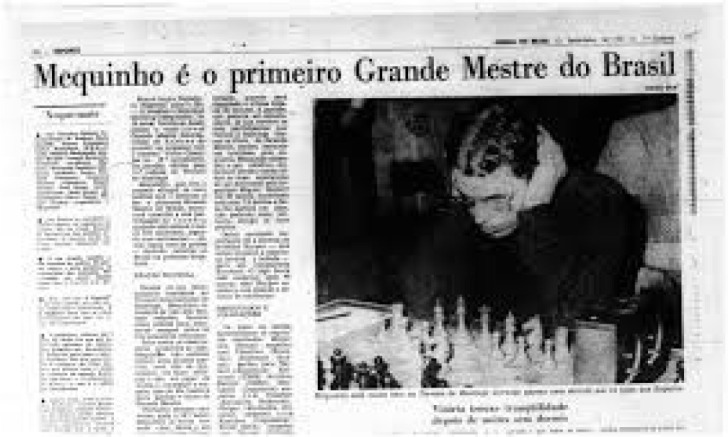
Mequinho is recognized as Grand Chess Master in Brazil, here playing in an international tournament.

Luiz Tavares was interested in underwater fishing, motorcycles, chess, and painting (Figure 6). Of all his hobbies, chess was his greatest passion. In addition to being a physician and splendid thoracic and cardiovascular surgeon, he was an outstanding chess player (Figure 7). He became president of the Brazilian Chess Confederation and Brazilian Chess Champion. He is considered a great protector and supporter of the World Grand Chess Master, Henrique da Costa Mecking (Mequinho), having accompanied his development from the beginning to occupy the 3^rd^ place in the world ranking (Figure 8). During an international chess tournament, he had a chance to meet Pelé, the world’s King of Soccer. Luiz Tavares asked him for an autograph for Mequinho, who was a very shy person. In response, he heard from Pelé: “but Doctor, how can I give an autograph to the best in the world using his head, if I am only the best using my feet”.

Luiz Tavares was also the founder of the Clube de Xadrez do Recife. He was runner-up in the Brazilian national chess tournament in 1956 and the Brazilian Champion in 1957, even though he was an “amateur” chess player. His brilliant intellectuality took him to the rank of a chess grandmaster, a thinker, who always hovered above the banality of everyday life.

### Tributes

**Fig. 9:**
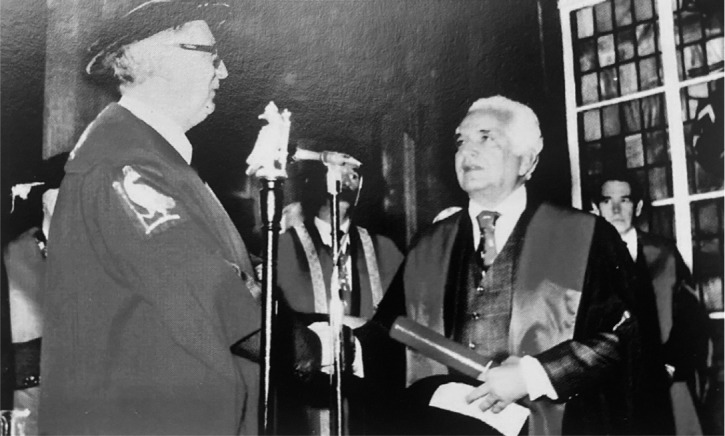
Luiz Tavares receiving the title of Fellow of the Royal College of Surgeons in London 1982, and Mr. Christopher Lincoln can be seen at the back right side of the photo.

Luiz Tavares received numerous honors from scientific societies in Brazil and abroad, but the most significant tribute came from England where he was recognized as an Honorary Member of the Royal College of Surgeons of England. The granting of this title for a Brazilian surgeon was unprecedented (Figure 9), and during the award ceremony, the distinguished English cardiac surgeon Mr. Christopher Lincoln compared him to the famous English cardiac surgeon Sir Lord Brock.
